# Method for Denoising the Vibration Signal of Rotating Machinery through VMD and MODWPT

**DOI:** 10.3390/s23156904

**Published:** 2023-08-03

**Authors:** Xiaolong Zhou, Xiangkun Wang, Haotian Wang, Zhongyuan Xing, Zhilun Yang, Linlin Cao

**Affiliations:** Mechanical Engineering College, Beihua University, Jilin City 132021, China; xlzhou1987@163.com (X.Z.);

**Keywords:** variational mode decomposition (VMD), maximal overlap discrete wavelet packet transform (MODWPT), mechanical vibration signal, gear, denoising

## Abstract

The vibration signals from rotating machinery are constantly mixed with other noises during the acquisition process, which has a negative impact on the accuracy of signal feature extraction. For vibration signals from rotating machinery, the conventional linear filtering-based denoising method is ineffective. To address this issue, this paper suggests an enhanced signal denoising method based on maximum overlap discrete wavelet packet transform (MODWPT) and variational mode decomposition (VMD). VMD decomposes the vibration signal of rotating machinery to produce a set of intrinsic mode functions (IMFs). By computing the composite weighted entropy (CWE), the phantom IMF component is then removed. In the end, the sensitive component is obtained by computing the value of the degree of difference (DID) after the high-frequency noise component has been decomposed through MODWPT. The denoised signal reconstructs the signal’s intrinsic characteristics as well as the denoised high-frequency IMF component. This technique was used to analyze the simulated and real-world signals of gear faults and it was compared to wavelet threshold denoising (WTD), empirical mode decomposition reconstruction denoising (EMD-RD), and ensemble empirical mode decomposition wavelet threshold denoising (EEMD-WTD). The outcomes demonstrate that this method can accurately extract the signal feature information while filtering out the noise components in the signal.

## 1. Introduction

Rotating machines are important types of equipment used in a variety of industrial applications. An unexpected fault in these machines could lead to significant economic losses and casualties. The vibration signal of rotating machinery carries information about its running state. Therefore, detection and analysis of the vibration signals of rotating machinery is usually the basis for understanding the service life and operating status of the equipment. It is an important basis used for preventive maintenance and fault diagnosis [1]. However, due to the interference of operating equipment and the field environment, various kinds of noise are inevitably introduced in the process of signal monitoring and acquisition. In order to ensure the authenticity of the measured signal and the effectiveness of subsequent signal feature extraction, it is particularly important to denoise the measured signal.

Currently, there are many denoising methods used in the analysis of vibration signal of rotating machinery, including the Fourier filter, wavelet transform (WT), and fast independent component analysis (FastICA) [2,3,4]. While these methods have achieved some success, they often encounter problems. Traditional denoising methods are mainly based on linear filtering [5], but vibration signals of rotating machinery are often non-linear and non-stationary due to environmental noise and varying operation states [6]. As a result, traditional methods are not ideal for handling such signals. WT also has some unavoidable defects, such as boundary distortion, energy leakage, and non-adaptiveness [7]. For the FastICA method, there is still a lot of residual noise, and the denoising signal needs to be selected from multi-dimensional input and output signals [8]. In 1998, a time–frequency analysis method named the Hilbert–Huang transform (HHT) was proposed by Huang [9]. HHT is derived from the principles of empirical mode decomposition (EMD) and the Hilbert transform. One of the main advantages of this technique is that it is a self-adaptive method, and the decomposition results are derived from the signal itself. Therefore, this method is widely used in the field of rotating machinery denoising research [10,11,12]. HHT may be a useful technique to extract the characteristics of non-stationary and non-linear signals. However, there are still some issues that need to be addressed to apply this method accurately. When decomposing signals with multiple frequency components, the presence of oscillations with very disparate amplitudes in a mode, or the presence of very similar oscillations in different modes, can cause “mode mixing” [13]. Additionally, the envelope estimation error of multiple frequency signals can cause end effects and pseudopulse phenomena. If these problems occur, the intrinsic mode function (IMF) component obtained through EMD does not have any real or physical meaning, and it becomes difficult to characterize signal characteristics. Therefore, finding an appropriate approach to avoid mode mixing, end effects, and pseudopulse phenomena is a significant objective for HHT method researchers.

Mode mixing is a major problem that affects the decomposition accuracy of EMD. To address this problem, researchers have proposed some new methods such as an improved EMD method based on singular value decomposition [14] or revised blind source separation [15], ensemble empirical mode decomposition (EEMD) [13], and complementary EEMD (CEEMD) [16]. Compared to other methods, EEMD is the most commonly used method for restraining the mode mixing problem [17] and for denoising rotating machinery fault signals [18,19,20]. EEMD performs decomposition over an ensemble of noisy copies of the signal and obtains the IMFs by averaging. However, EEMD introduces new difficulties. Due to its algorithm, the decomposition of EEMD is incomplete, and the selection of noise standard deviation may result in a different number of IMFs.

In 2014, Dragomiretskiy proposed a variable-scale non-stationary signal analysis method and named it variational mode decomposition (VMD) [21]. This method is capable of decomposing a complex signal into the sum of multiple single-component amplitude-modulated and frequency-modulated signals. The number of modes in the decomposition process can be adaptively determined. VMD effectively avoids the problem of mode mixing and exhibits better noise robustness, so it has gained much attention by researchers since it was proposed [22,23,24]. In this method, the key step in the decomposition is to find the appropriate parameters *K* (the number of mode functions) and *α* (the penalty factor), which affect the decomposition precision. Some researchers have used intelligent search algorithms to select *K* and *α* for higher precision [25,26]. Intelligent search methods can solve complex problems without prior knowledge, but they require significant computing time and can be difficult to use for actual detection. At the same time, filtering out the noise component is vital to ensure the accuracy of signal feature extraction, but it can be difficult to accurately select the illusive IMFs due to the presence of noise [27].

Maximal overlap discrete wavelet packet transform (MODWPT) [28] can be regarded as a modified discrete wavelet transform (DWT), which is a highly redundant non-orthogonal WT, and has no requirement for the sample size *N*. Unlike the traditional DWT method, MODWPT has the advantages of the translation invariance of wavelet coefficients and scale coefficients. All resolution layers maintain the same time resolution with no phase distortion, making MODWPT very suitable for processing non-linear and non-stationary signals. At the same time, discrete wavelet packet transform (DWPT) can effectively compensate for the defect where the discrete wavelet transform cannot further decompose high-frequency bands. MODWPT not only has all the advantages of DWPT, but can also further decompose high-frequency bands, thereby improving frequency resolution. Table 1 lists the characteristics of common signal decomposition methods.

Based on VMD and MODWPT, this paper proposes a novel signal denoising method. This method is used to denoise simulated signals and measured rotating machinery fault signals. The main contributions of this paper are summarized as follows:

(1) The number *N* of effective center frequencies and minimum value of multi-scale dispersion entropy (MDE) are proposed to determine the key parameters *K* and *α* of VMD;
(2) The comprehensive weighted entropy (CWE) of IMF components is used to select the sensitive IMFs of VMD, containing the signal feature information. The high-frequency noise IMFs are decomposed through MODWPT and the noise components are filtered out by calculating the difference degree (DID). The non-stationary rotating machinery signals are denoised by reconstructing the sensitive components of VMD and MODWPT;
(3) The effectiveness and performance of the proposed method is verified by analyzing the simulated signal and real experimental gear fault signal. These results are compared with wavelet threshold denoising (WTD), EMD reconstruction denoising (EMD-RD), and EEMD wavelet threshold denoising (EEMD-WTD) to evaluate the performance.

The rest of this paper is organized as follows. Section 2 provides a brief overview of the fundamental theories. Section 3 describes the calculation process for the proposed denoising method. Section 4 provides a simulated signal example to demonstrate the reliability and effectiveness of the proposed method. Section 5 discusses the use of this method in fault detection and denoising of vibration signals from rotating machinery. Section 6 contains the paper’s main conclusions.

## 2. Brief Introduction of Basic Theories

### 2.1. VMD Method

The VMD method can decompose a signal *f* into a series of modal functions *u*_1_, *u*_2_, …, *u_k_* according to the preset scale parameter *K*. The resulting constrained variational problem is the following:

(1) $$\left.\begin{array}{c} \min_{u_{k},\omega_{k}}\;\left(\sum_{k=1}^{K}\;{∥\partial_{t}\left[\left(\delta (t)+\frac{j}{\pi t}\right)*u_{k}(t)\right]e^{-j\omega_{k}t}∥}_{2}^{2}\right)\\ \sum_{k=1}^{K}\;u_{k}=f \end{array}\right\}$$

where {*u_k_*}: = {*u*_1_, …, *u_K_*} and {*ω_k_*}: = {*ω*_1_, …, *ω_K_*} are shorthand notations for the set of all modes and their center frequencies, respectively. *∂_t_* is the partial derivative of the time t for the function, *δ*(*t*) is the unit pulse function, and ∗ represents the convolution operation.

We introduce the augmented Lagrangian *ζ* as given below, and the constrained variational problems are transformed into unconstrained variational problems:

(2) $$\zeta (u_{k},\omega_{k},\lambda )=\alpha {\sum_{k=1}^{K}\left\|\partial_{t}\left[\left(\delta (t)+\frac{j}{\pi \;t}\right)*u_{k}(t)\right]e^{-j\omega_{k}t}\right\|}_{2}^{2}+{\left\|f(t)-\sum_{k=1}^{K}u_{k}(t)\right\|}_{2}^{2}+\langle \lambda (t),f(t)-\sum_{k=1}^{K}u_{k}(t)\rangle$$

The solution to the original minimization problem is now found as the saddle point of the augmented Lagrangian in a sequence of iterative sub-optimizations called the alternate direction method of multipliers. The multipliers include $u_{k}^{n+1}$, $\omega_{k}^{n+1}$, and $\lambda^{n+1}$, where $u_{k}^{n+1}$ is the modal function at the (*n* + 1)th cycle, $\omega_{k}^{n+1}$ is the center frequency of the power spectrum of the current modal function, and $\lambda^{n+1}$ is the multiplication operator at the (*n* + 1)th cycle.

Then, the modal component *u_k_* and the center frequency *ω_k_* are derived:

(3) $${\hat{u}}_{k}^{n+1}(\omega )=\frac{\hat{f}(\omega )-\sum_{i\neq k}{\hat{u}}_{i}(\omega )+\frac{\hat{\lambda }(\omega )}{2}}{1+2\alpha {\left(\omega -\omega_{k}\right)}^{2}}$$

(4) $$\omega_{k}^{n+1}=\frac{\int_{0}^{\infty }\omega {\left|{\hat{u}}_{k}(\omega )\right|}^{2}d\omega }{\int_{0}^{\infty }{\left|{\hat{u}}_{k}(\omega )\right|}^{2}d\omega }$$

where ${\hat{u}}_{k}^{n+1}$, $\hat{f}$, and ${\hat{\lambda }}^{n+1}$ represent the Fourier transform corresponding to $u_{k}^{n+1}$, f, and $\lambda^{n+1}$, respectively.

For convergence accuracy *e* > 0, when Equation (5) is satisfied, the decomposition stops. Thus, the final modal component ${\hat{u}}_{k}^{}$ and its center frequency *ω_k_* are given as

(5) $$\sum_{k=1}^{K}\left({{\left\|{\hat{u}}_{k}^{n+1}-{\hat{u}}_{k}^{n}\right\|}_{2}^{2}/\left\|{\hat{u}}_{k}^{n}\right\|}_{2}^{2}\right)<e$$

### 2.2. VMD Key Parameter Selection Method

The preset scale parameter *K* and penalty factor *α* directly affect the accuracy of the VMD decomposition results. Although the current intelligent search algorithm can optimize the parameter values given above, such methods take a long time and it is difficult to achieve the purpose of actual detection. Therefore, this paper proposes an efficient and simple method of VMD decomposition key parameter selection.

#### 2.2.1. Selection of *K*

The center frequency of each IMF component obtained through VMD is distributed from a low to a higher level. If the optimal value of *K* is obtained, this means that the center frequency distribution between adjacent IMF components is reasonable and the final result value will not be similar or mixed.

Therefore, researchers currently use the center frequency observation method to determine the optimal value of *K* [29,30]. However, these methods lack quantitative judgment standards and universality in the actual signal analysis process.

**Figure 1:**
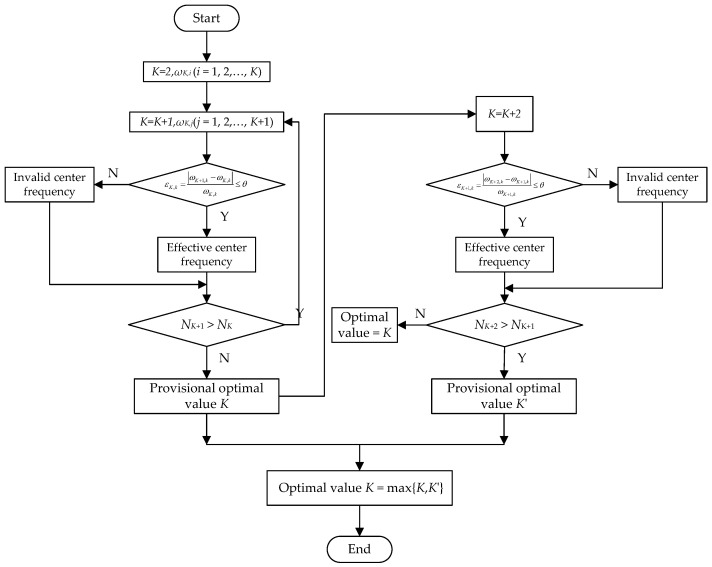
The algorithm flow chart of selection of *K*.

From Equation (5), it can be seen that VMD uses the frequency domain change of the IMF component during two consecutive cycles as the iterative constraint condition to determine when to stop the decomposition. To solve this problem, this paper proposes an algorithm to select *K* according to the number *N* of effective center frequencies by taking the change in the center frequency value between two adjacent decomposition processes of the same order modal component as the judgment condition. Figure 1 shows the algorithm flow chart. The specific process of the algorithm is as follows:

Step 1: Initialize the value of *K*, let *K* = 2.
Step 2: Decomposed the signal through the VMD method and obtain *K* order IMF components and the center frequency of each IMF component *ω_K_*_,*i*_ (*i* = 1, 2, …, *K*).
Step 3: Let *K* + 1 and perform VMD decomposition on the signal again to obtain *K* + 1 order IMF components and the center frequency of each IMF component *ω_K_*_+1,*j*_ (*j* = 1, 2, …, *K* + 1).
Step 4: According to Equation (6), calculate the accuracy of the center frequency of each IMF component under the same order and different decomposition times *ε_K,k_* (*k* = 1, 2, …, *K*):
  (6) $$\varepsilon_{K,k}=\frac{\left|\omega_{K+1,k}-\omega_{K,k}\right|}{\omega_{K,k}}$$
  where *ω_K+_*_1*,k*_ is the center frequency of the (*K* + 1)th order IMF component obtained through VMD and *ω_K,k_* is the center frequency of the *K*-th order IMF component obtained through VMD.
Step 5: Determine the size of the judgment accuracy *ε_K,k_* and the accuracy threshold *θ*. If the value of *θ* is too small, it is difficult to effectively distinguish the frequency domain characteristics between components. If the value of *θ* is too large, it is also difficult to accurately reflect the frequency domain difference between each component because the vibration signal of rotating machinery is often composed of multiple frequency components. Therefore, within a reasonable range, the value of *θ* selected should be as large as possible. Based on [31] and a large number of tests, the value of *θ* is set to 0.15. If *ε_K_*_,*k*_ ≤ *θ*, it is considered as the effective center frequency. If *ε_K_*_,*k*_ > *θ*, it is regarded as the invalid center frequency. Let *N_K_*_+1_ and *N_K_* be the number of consecutive effective center frequencies obtained from the IMF1 component, whereas *K* + 1 and *K* are used to determine the number of mode functions.
Step 6: If *N_K_*_+1_ > *N_K_*, the VMD decomposition is considered incomplete. Then, repeat step 3–step 5.
Step 7: If *N_K_*_+1_ ≤ *N_K_*, the number of consecutive effective center frequencies no longer increases, so it is considered that there is suspected over-decomposition, and thus the provisional optimal value is *K*.
Step 8: Consider that the current provisional optimal value is *K*. If the signal to be decomposed contains multiple and different frequency components, there is still the possibility of under-decomposition. To avoid the above problems, let *K* = *K* + 2 and repeat step 3–step 5. If *N_K_*_+2_ ≤ *N_K_*_+1_, the optimal preset scale is *K*; if *N_K_*_+2_ > *N*_K+1_, then repeat step 3 to step 7 and obtain the second provisional value. If *N_K_*_+3_ ≤ *N_K_*_+2_, the second tentative optimal value is *K* + 2, and the parameter corresponding to max{*N_K_*, *N_K_*_+2_} is the optimal value.

#### 2.2.2. Selection of *α*

*α* is another important parameter to be set during the process of VMD decomposition, which determines the bandwidth of IMFs. The larger the *α* value, the smaller the bandwidth of the IMF component. As the VMD algorithm shows good noise robustness, after the vibration signal of rotating machinery is decomposed, the components of background interference and environmental noise in the signal should be filtered out. Additionally, the reconstructed signal should contain more feature information and demonstrate strong regularity and self-similarity [6,21].

Multi-scale dispersion entropy (MDE) is an important factor in evaluating the complexity of non-stationary signals [32,33]. MDE is based on dispersion entropy (DE); compared with multi-scale sample entropy (MSE) and scale arrangement entropy (MAE), MDE effectively simplifies the calculation process, enhances the accuracy of feature extraction, is less affected by abrupt signals, and has higher algorithm stability. Based on the analysis above, if VMD is optimized with an optimal *α*, it will enhance the regularity and self-similarity of a rotating machinery’s fault signal. Consequently, the MDE value of the reconstructed signal will be minimized.

For a signal {*x*(*t*), *t* = 1, 2, …, *T*}, a compound coarse-grained signal is used and the *k*-th coarse-grained sequence under the set scale factor *τ* is $x_{k}^{\tau }$. The coarse-grained signal can be obtained as

(7) $$x_{k,j}^{\left(\tau \right)}=\frac{1}{\tau }\sum_{i=k+(j-1)\tau }^{k+j\tau -1}x(t)$$

where 1 ≤ *j* ≤ *T*/*τ*, 1 ≤ *k* ≤ *τ*, $x_{k}^{\tau }=\left\{x_{k,1}^{\left(\tau \right)},x_{k,2}^{\left(\tau \right)},\cdots \right\}$.

Calculate DE with each scale factor *τ* of the coarse-grained signal, and MDE can be formed as

(8) $$MDE(X,m,c,\tau )=\frac{1}{\tau }\sum_{k=1}^{\tau }DE(x_{k}^{\tau },m,c,d)$$

where *m* is the dimension of the embedded vector, *c* is the number of categories, and *d* is the time delay.

After selecting *K*, calculate the MDE of the reconstructed signal obtained through VMD of the penalty factor *α* in different value ranges. The selection steps for parameter *α* are outlined as follows:

Step 1: Initialize the value of *α*; let *α* = 100.
Step 2: Calculate the MDE of the reconstructed signal obtained through VMD and obtain MDE1.
Step 3: To ensure the bandwidth of IMF components, let *α* = 200, calculate the MDE of the reconstructed signal obtained by VMD, and obtain MDE2.
Step 4: Repeat step 3 to calculate the MDE of the reconstructed signal obtained through VMD under *α* = 300–5000 [21] and form {MDE*i*}(*i* = 1, 2, …, 50).
Step 5: Select *α* corresponding to min{MDE*i*} as the optimal value.

### 2.3. Illusive IMF Component Selection Algorithm Based on Comprehensive Weighted Entropy

At present, there are few methods for identifying illusive IMF components. Existing methods often rely solely on signal characteristics in the time or frequency domain, making it difficult to comprehensively and accurately judge. Therefore, this paper proposes an illusive IMF identification method based on CWE.

In rotating machinery equipment, when a fault occurs, the energy in the fault signal frequency band will significantly differ, and energy entropy (EE) can effectively describe the change in signal energy with frequency distribution [34]. Power spectrum entropy (PSE) is a nonlinear characteristic quantity that characterizes the complexity of the signal. It can also characterize the distribution of the vibration spectrum type of the signal in the frequency domain [35]. The time–amplitude–frequency product can effectively reflect the change relationship between time, energy, and frequency and highlight the local characteristics of the signal, and has good time–frequency resolution. Time–amplitude–frequency product entropy (TPE) fully utilizes the advantages of entropy in signal information evaluation while avoiding the influence of similarity between fault features. It can more effectively depict the internal characteristics of the signal and has more accurate signal feature extraction ability [36]. The specific process of the illusive IMF component selection method based on CWE is as follows:

Step 1: For each IMF component {*u_i_*(*t*), *t* = 1, 2, …, *T*}, calculate the energy *e*(*u_i_*), power spectrum *p*(*u_i_*), and time–amplitude–frequency product *q*(*u_i_*).
  (9) $$\left\{\begin{array}{l} e(u_{i})=\int_{0}^{T}u_{i}{\left(t\right)}^{2}dt\\ p(u_{i})=\frac{1}{T}{\left|X(u_{i})\right|}^{2}\\ q(u_{i})=\sum_{j=1}^{m}S_{j} \end{array}\right.$$
  where *X*(*u_i_*) is the discrete Fourier transform of each sample in signal component *u_i_*(*t*) and *S_j_* (*j* = 1, 2, …, *m*) is the energy of each time–frequency plane of the Hilbert spectrum (the entire time–frequency plane is divided into m blocks, on average).
Step 2: Normalize the energy, power spectrum, and time–amplitude–frequency product of all IMF components.
  (10) $$\left\{\begin{array}{l} e(i)=e(u_{i})/\sum_{i=1}^{K}e(u_{i})\\ p(i)=p(u_{i})/\sum_{i=1}^{K}p(u_{i})\\ q(i)=q(u_{i})/\sum_{i=1}^{K}q(u_{i}) \end{array}\right.$$
  where *K* is the number of IMF components decomposed through VMD.
Step 3: Calculate the entropy increment of each IMF component.
  (11) $$\left\{\begin{array}{l} EE_{i}=-g(i)\log_{2}(g(i))\\ PSE_{i}=-p(i)\log_{2}(p(i))\\ TPE_{i}=-q(i)\log_{2}(q(i)) \end{array}\right.$$
Step 4: Obtain the CWE value of each IMF component according to Equation (12).
  (12) $$CWE_{i}=\delta \times EE_{i}+\beta \times PSE_{i}+\gamma \times TPE_{i}$$
  where *α*, *β,* and *γ* are weighting coefficients and *δ* + *β* + *γ* = 1.
Step 5: Calculate the principal component factor *λ_i_* based on CWE.
  (13) $$\lambda_{i}=\frac{CWE_{i}-\min(CWE)}{\max(CWE)-\min(CWE)}$$
Step 6: Arrange all components in the order of *λ_i_* from large to small to obtain the component sequence $\left\{\lambda^{\prime },\lambda_{1}^{\prime }>\lambda_{2}^{\prime },\cdots ,\lambda_{n-1}^{\prime }>\lambda_{n}^{\prime }\right\}$.
Step 7: Calculate the *λ_i_* difference of two adjacent components.
  (14) $$d_{i}=\lambda_{i}^{\prime }-\lambda_{i+1}^{\prime }$$
Step 8: Find the index *m* of the maximum difference. Then, the first *m* components of sequence {*λ*′} contain the main characteristic information of the signal and represent the sensitive IMF component. The IMF component corresponding to *λ_i_* = 0 is the noise or illusive component, which should be eliminated.

**Figure 2:**
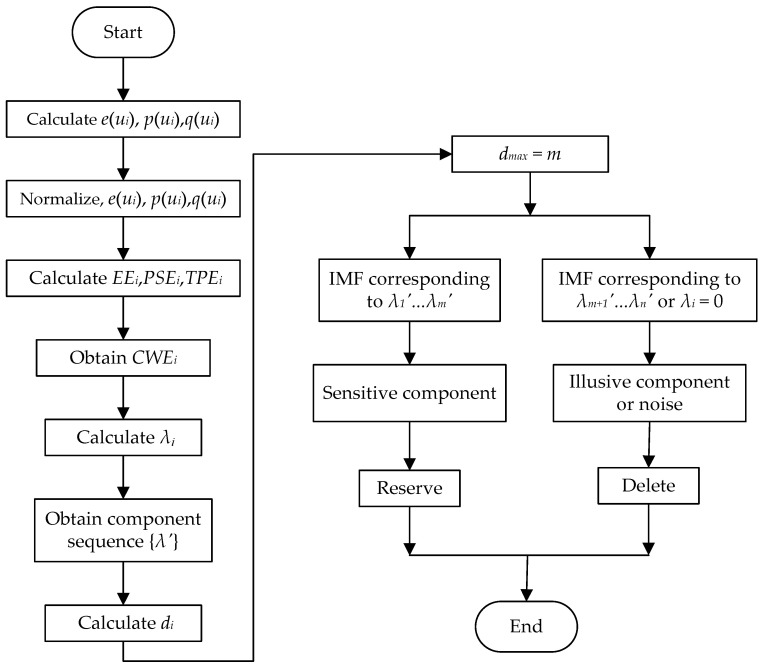
The algorithm flow chart of selection of illusive IMF component.

The proposed method comprehensively considers the relationship between each IMF component and the decomposed signal in the time domain, frequency domain, and time–frequency domain. This method can enhance the main components of the signal, weaken the interference components irrelevant to the signal characteristics, and ensure the purity of the signal characteristics. Moreover, it avoids the need to propose a discrimination threshold, and the accuracy of the calculation results is more scientific and reliable. Figure 2 shows the algorithm flow chart.

### 2.4. MODWPT Method

Generally, the IMF component that characterizes the high-frequency noise component contains not only noise but also useful information about the signal [37]. If these high-frequency components are directly filtered as noise interference, key information in the signal may be lost, reducing the accuracy of the signal feature extraction. In order to enhance the signal-to-noise ratio and the denoising effect, the MODWPT method is used to decompose the IMF component that characterizes high-frequency noise.

The decomposition coefficient of MODWPT can be represented by *W_j_*_,*n*_ = {*W_j_*_,*n*,*t*_, *t* = 0,…, *N* − 1}, where *j* is the number of decomposition layers, *n* can be regarded as a frequency index that changes with *j*, and the decomposition coefficient of MOWDPT can be calculated as follows:

(15) $$W_{j,n,t}=\sum_{l=0}^{l-1}r_{n,l}W_{j-1,[n/2],(t-2^{j-1}l)\mathrm{mod}N}$$

where if *n* mod4 = 0 or 3, $r_{n,l}=\{{\tilde{g}}_{l}\}$, if *n* mod4 = 1 or 2, and $r_{n,l}=\{{\tilde{h}}_{l}\}$. Among them, ${\tilde{g}}_{l}$ and ${\tilde{h}}_{l}$ represent the scale filter and wavelet filter of MODWT, respectively.

When a rotating machine malfunctions, usually only a certain component malfunctions, such as the rotor, gear, or bearing. Therefore, only a small part of the vibration information can be used to indicate the failure of the rotating machinery after using MOTWPT to decompose the high-frequency noise components containing some information on the fault characteristics. The fault characteristics are concentrated only on a certain component. At the same time, there is a certain correlation between the fault characteristics and the signal characteristic information under normal conditions. The noise interference component contains a few normal signal characteristics; hence, there is a large degree of information difference between the noise interference component decomposed through the MODWPT and the normal signal.

The model formula of the DID [38] can be expressed as follows:

(16) $$DID=[\alpha_{1}\left(\left|\mu_{n}-\mu_{un}\right|+\left|\delta_{n}+\delta_{un}\right|\right)\frac{\left|\mu_{n}-\mu_{un}\right|}{\delta_{n}^{2}+\delta_{un}^{2}}+\alpha_{2}\ln\left(\frac{\delta_{n}^{2}+\delta_{un}^{2}}{2\delta_{n}\delta_{un}}\right)]\sqrt{\sum \left(y_{i}-x_{i}\right)}$$

where *µ_n_*(*i*), *δ_n_*(*i*), *µ_un_*(*i*), and *δ_un_*(*i*) are the mean and standard deviations of the normal and fault signals, and *x_i_* and *y_i_* are normal and fault signals.

By combining the DID technique and the MODWPT method, the noise interference components in the high-frequency IMF component can be effectively filtered out.

## 3. The Signal Denoising Method Based on VMD and MODWPT

The vibration signal of rotating machinery is always mixed with different noises during the acquisition process, which affects the accuracy of the signal feature extraction. The steps of the denoising method based on VMD and MODWPT are as follows:

Step 1: Use the VMD method to decompose the vibration signal of rotating machine, and determine the optimal value of *K* based on the successive number of effective center frequencies.
Step 2: After selecting *K*, calculate the MDE of the reconstructed signal obtained through VMD of the penalty factor *α* in different value ranges. Select *α* corresponding to min{MDE} as the optimal value.
Step 3: Calculate the principal component factor *λ_i_* based on the CWE of each IMF component, determine the illusive IMF components, and obtain the preliminary denoising signal *x*′(*t*).
Step 4: Based on the center frequency of the illusive IMF components, decompose the IMF components (*λ_i_* ≠ 0), which represent the high-frequency illusive component, through MODWPT. Calculate the value of the DID between the component obtained through MODWPT and the normal signal, filter out the noise components, and determine the components containing the characteristic information of the signal to further improve the denoising effect.
Step 5: Reconstruct the IMF component denoised through MOWDPT and the IMF components of each order characterizing the signal’s characteristics to form a denoised signal *x*″(*t*).

**Figure 3:**
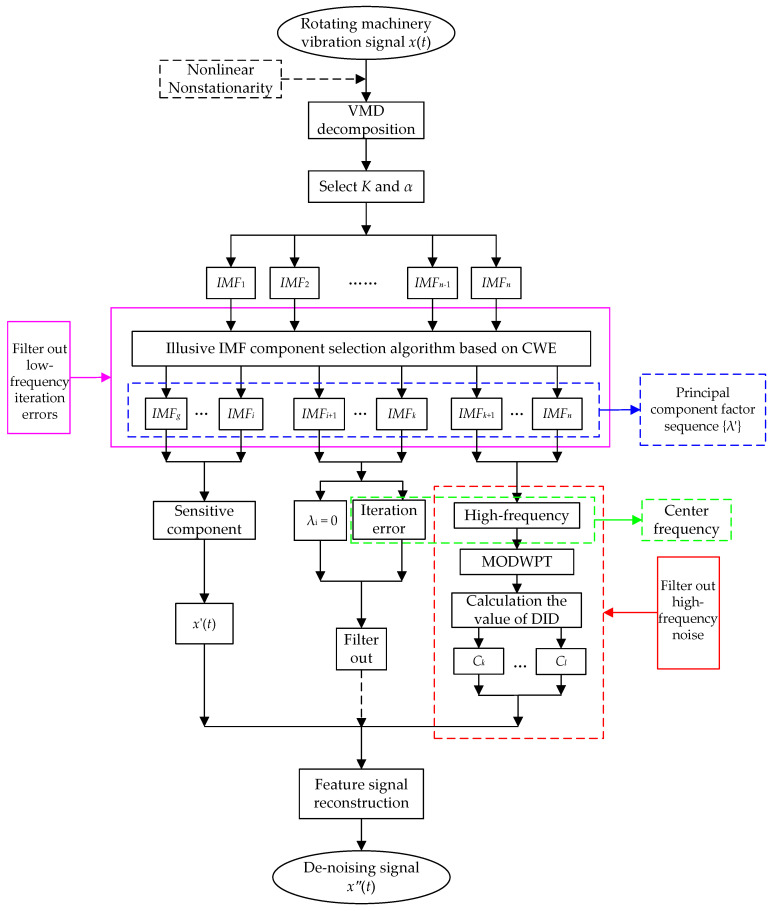
Algorithm flow chart.

The algorithm flow chart is shown in Figure 3.

## 4. Simulation Analysis

Usually, the vibration signal of rotating machinery can be simulated using the superposition of the amplitude modulation, frequency modulation and the Gaussian white noise [39]. Based on the research findings of Sun et al. [39], the following analogue signals are established to more realistically simulate the multi-component vibration signals of rotating machinery:

(17) $$\left\{\begin{array}{l} z(t)=x(t)+n(t)\\ x(t)=x_{1}(t)+x_{2}(t)+x_{3}(t)\\ x_{1}(t)=0.3[1+\sin(60\pi t)]\cos[400\pi t+2\cos(60\pi t)]\\ x_{2}(t)=0.2[1+\sin(60\pi t)]\cos[1200\pi t+2\cos(60\pi t)]\\ x_{3}(t)=0.1[1+\sin(60\pi t)]\cos[2000\pi t+4\cos(60\pi t)] \end{array}\right.$$

where *n*(*t*) is the Gaussian noise signal. It is obtained by using the ‘randn’ function in MATLAB software (version 2016a) and its SNR is −9.5 dB.

**Figure 4:**
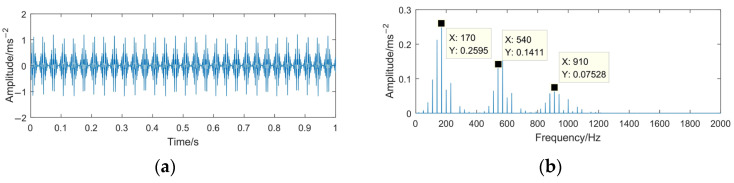
Time domain waveform and frequency spectrum of the simulated signal *x*(*t*). (**a**) Waveform of *x*(*t*); (**b**) spectrum of *x*(*t*).

**Figure 5:**
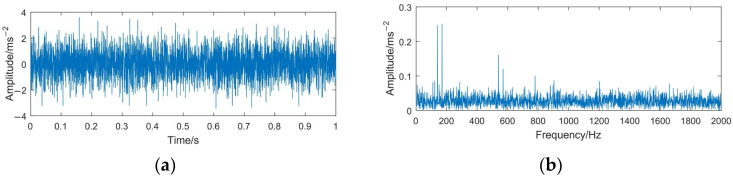
Time domain waveform and frequency spectrum of the simulated signal *z*(*t*). (**a**) Waveform of *z*(*t*); (**b**) spectrum of *z*(*t*).

The sampling frequency is 4096 Hz and the sampling time is 1 s. The time domain waveforms of the simulation signals *x*(*t*) and *z*(*t*) are shown in Figure 4 and Figure 5.

From Figure 4, it can be seen that the simulated signal contains three main center frequencies: 170 Hz, 540 Hz, and 910 Hz. When the rotating machinery signal is weak or covered by strong background noise, the signal characteristics (impact property and center frequency) may not appear apparently. Comparing Figure 4 with Figure 5, it can be seen that the periodic impact components in the simulated signal are completely submerged by noise, the center frequency 910 Hz is difficult to distinguish, and the high-frequency part is severely affected by noise, with many interference spectral lines appear in this part.

In order to denoise the simulated signal, the VMD-MODWPT method is used. Firstly, the simulated signal *z*(*t*) is decomposed through VMD. The center frequency of each IMF component of *z*(*t*) is obtained under different *K* values. In order to obtain the optimal value of *K*, the judgment accuracy of each IMF component corresponding to different *K* values is calculated according to the method proposed in this paper. The calculation results are shown in Table 2. Table 2 shows that *N*_4_ = 4 > *N*_5_ = 1, so *K* = 4 can be tentatively set as the optimal value and *N*_6_ = *N*_5_ = 1. Therefore, the optimal number of mode functions is *K* = 4.

This method is applied to the rolling bearing vibration data of Case Western University in [40,41]. The analysis proves the effectiveness of the algorithm for selecting the value of *K* based on the number *N* of the effective center frequencies. Due to space limitations, only Table 1 is listed in [40]. The decision accuracies of each IMF component of the bearing inner ring fault signal corresponding to different *K* values are provided in Table 3.

From Table 3, it can be seen that *N*_2_ = 1 > *N*_3_ = 0, *K* = 2 can be tentatively set to the optimal value. At the same time, *N*_4_ = 4 > *N*_5_ = 3 and *N*_4_ > *N*_2_. Therefore, the optimal value of the preset scale is *K* = 4. The above analysis verifies the effectiveness of the proposed method; it also proves the importance of step 8 in the algorithm for the signals composed of multiple frequency components. And if *θ* > 0.15 (for example *θ* = 0.16), this may cause *N*_5_ = 6 > *N*_4_ = 4, resulting in excessive selection of *K* and causing over-decomposition and modal mixing problems. Further, the analysis of the measured rolling bearing and the gearbox fault signals from [42,43] proves the effectiveness and practicality of this method.

**Figure 6:**
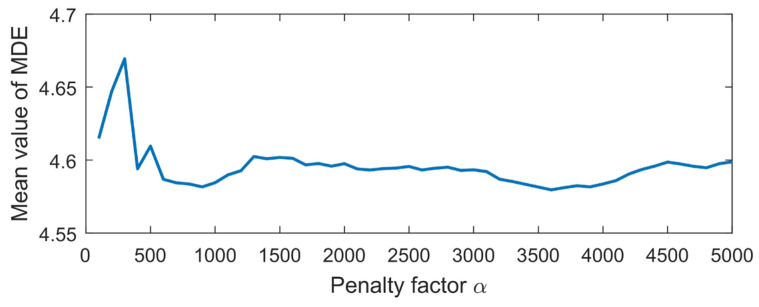
The mean MDE value of the reconstruction signal under different *α*.

When *K* = 4, the penalty factor *α* is calculated in different ranges, and the mean MDE value of the reconstruction signal is shown in Figure 6. During MDE calculation, the dimension of the embedded vector *m* = 3, the number of categories *c* = 6, the time delay *d* = 1, and the scale factor *τ*_max_ = 10.

In Figure 6, when *α* = 3600, the mean MDE value of the reconstructed signal after VMD decomposition is the smallest. This indicates that the impact component related to the fault feature in the reconstructed signal contains the most, and also shows strong regularity and self-similarity, so *α* = 3600 is taken to decompose the simulation signal.

**Figure 7:**
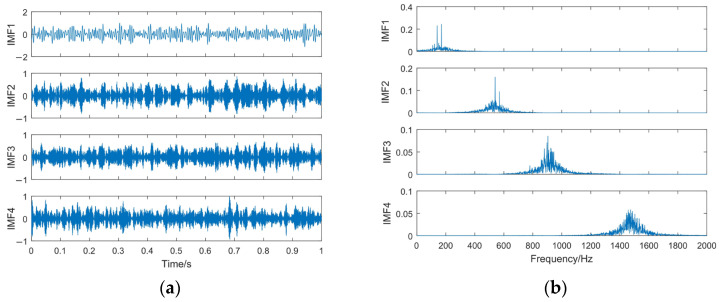
The VMD decomposition results. (**a**) Time domain waveform of IMFs; (**b**) spectrum of IMFs.

According to Table 1 and Figure 4, *K* = 4 and *α* = 3600 are used in VMD. The VMD decomposition results of the simulated signal *z*(*t*) and the spectrum of each IMF component are shown in Figure 7.

It can be seen from Figure 7 that the VMD decomposition results are reasonable, in which the IMF1–IMF3 are amplitude modulation–frequency modulation signals, whereas the IMF4 is a high-frequency Gaussian white noise component. The frequency of the IMF1–IMF3 components is mainly concentrated near the center frequency. It is verified that the parameter selection method can effectively suppress the modal aliasing problem generated in the decomposition process. At the same time, it can reduce the information leakage between the modal components. However, it can also be seen that the frequency characteristics of the IMF3 component are hidden by noise, so its feature information must be effectively extracted and restored.

**Figure 8:**
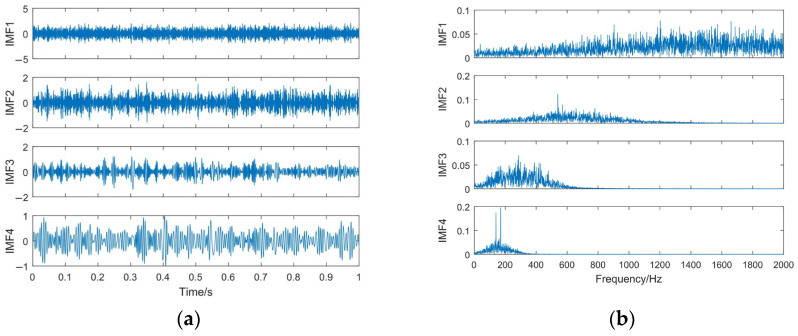
The EMD decomposition results. (**a**) Time domain waveform of IMFs; (**b**) spectrum of IMFs.

**Figure 9:**
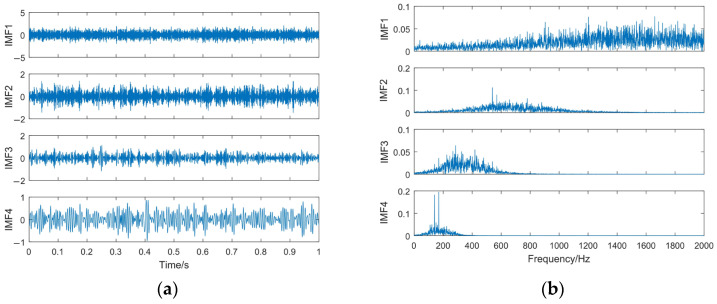
The EEMD decomposition results. (**a**) Time domain waveform of IMFs; (**b**) spectrum of IMFs.

To compare the decomposition effect, the EMD method [12] and the EEMD method are used to decompose the simulated signal *z*(*t*). The decomposition results and the spectrum of each IMF component are shown in Figure 8 and Figure 9. An ensemble size of *I* = 100 and standard deviation *ε*_0_ = 0.2 are used in EEMD [13,44]. Further, for the sake of convenience or convenience of contrast, EMD and EEMD take the first to fourth IMF for analysis.

According to the information given in Figure 6 and Figure 7, EEMD suppresses the modal mixing problem to a certain extent. However, it can also be seen that the decomposition result is not ideal. EMD and EEMD obtain many iteration error components, especially for the IMF1, in which almost all the frequency components exist in its entire frequency band. This inevitably affects the accuracy of the subsequent signal feature extractions.

**Figure 10:**
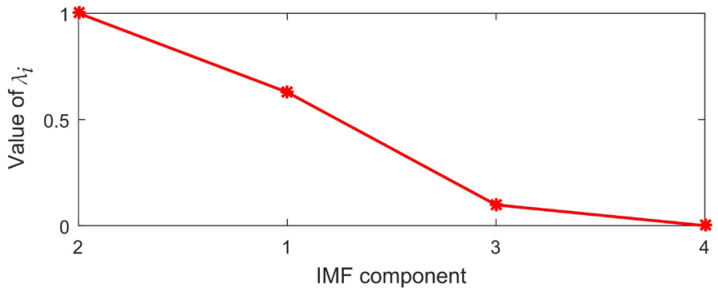
The principal component factor *λ_i_* of each IMF decomposed through VMD.

The CWE value of each IMF component decomposed through VMD is calculated and the principal component factor *λ_i_* is obtained based on CWE. The *λ_i_* of each IMF component is show in Figure 10. During CWE calculation, *δ* = 0.2 and *β* = *γ* = 0.4.

From Figure 10, it can be seen that the maximum difference of the principal component factors *λ_i_* is between IMF1 and IMF3, so the first two components (IMF2 and IMF1) after reordering are the components, which containing the main characteristics of the signal. Therefore, the illusive IMF component selection algorithm based on CWE can properly remove the interference components. According to the previous analysis, IMF3 is a high-frequency amplitude modulation–frequency modulation component of the simulation signal, whose characteristics are hidden by noise. In order to ensure the denoising effect, it is necessary to extract its feature. For the IMF4 component, its principal component factor *λ*_4_ *=* 0, so this component is recognized as the noise component, which shall be eliminated.

**Figure 11:**
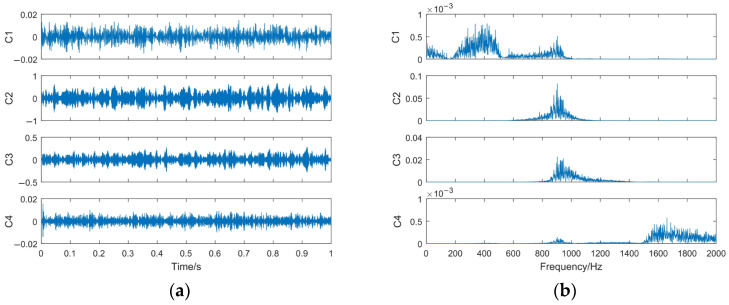
The MODWPT decomposition results of IMF3. (**a**) Time domain waveform of components; (**b**) spectrum of components.

In the decomposition process, the IMF3 component is decomposed through MOWDPT. Based on [28], during the decomposition process, the Fejer–Korovkin wavelet filter with length *L* = 22 and decomposition layer number *J* = 2 is selected. The decomposition results are shown in Figure 11.

In Figure 11, the impact characteristics of the C2 and C3 components are obvious and the peak value of the spectral line at the central frequency of the C2 and C3 components is large. In order to determine the component containing the characteristic information of the signal, the value of the DID between each component and the normal signal is calculated. The results are shown in Table 4. In DID calculation, *α*_1_ = 100, *α*_2_ = 0.1.

**Figure 12:**
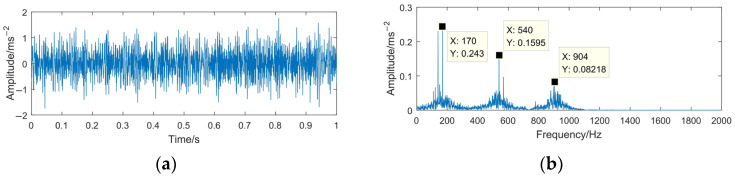
Time domain waveform and frequency spectrum of the denoising signal based on VMD-MODWPT. (**a**) Waveform of denoising signal; (**b**) spectrum of denoising signal.

It can be seen from Table 3 that the value of the DID between the C2 component and the normal signal is the smallest. This indicates that the above components contain lots of information on the normal characteristics. Therefore, this component is judged as the high-frequency denoising component, and the denoising simulation signal is reconstructed using the IMF1 and IMF2 obtained through VMD and the C2 component of IMF3 obtained through MODWPT. The time domain waveform and the spectrum of the denoising signal are shown in Figure 12.

Comparing Figure 4 with Figure 12, it can be seen that after processing with the proposed VMD-MODWPT method, most of the useless interference and noise components in the simulated signal *z*(*t*) are filtered out effectively, and the signal time domain waveform and the spectrum effectively highlight the characteristic information of the signal *x*(*t*).

**Figure 13:**
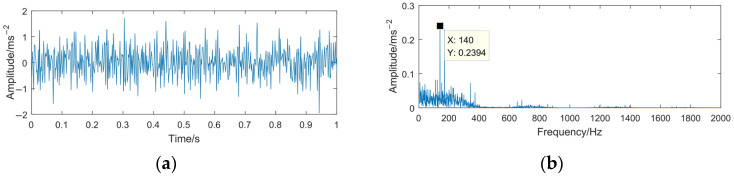
The WTD denoising result of *z*(*t*). (**a**) Time domain waveform of *z*(*t*); (**b**) spectrum of *z*(*t*).

**Figure 14:**
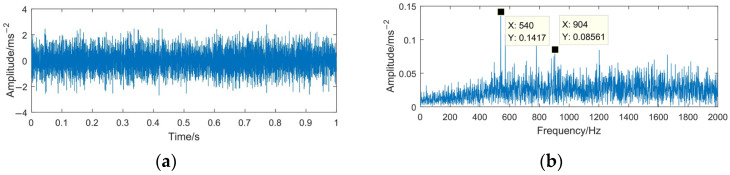
The EMD-RD denoising result of *z*(*t*). (**a**) Time domain waveform of *z*(*t*); (**b**) spectrum of *z*(*t*).

**Figure 15:**
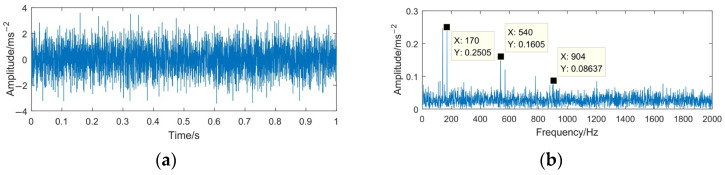
The EEMD-WTD denoising result of *z*(*t*). (**a**) Time domain waveform of *z*(*t*); (**b**) spectrum of *z*(*t*).

To highlight the effectiveness and superiority of the proposed method, WTD, EMD-RD [45], and EEMD-WTD [37] were used to deal with the simulation signal z(t). The denoising results of different methods are shown in Figure 13, Figure 14 and Figure 15. Based on [13,44,46], WTD adopts the ‘db3’ wavelet basis function for three-layer decomposition and the ‘Rigrsure’ rule for soft threshold denoising; for EEMD-WTD, an ensemble size of *I* = 100 and standard deviation *ε*_0_ = 0.2 are used in EEMD.

It can be seen from Figure 13, Figure 14 and Figure 15 that the denoising effect of the above method is not ideal, because a large amount of noise remains in the signal even after denoising, which inevitably affects the subsequent signal analysis effect. For the WTD method, the main characteristic information of the simulated signal is also filtered. The center frequencies 540 Hz and 910 Hz do not show spectral lines in the spectrum. For the EMD-RD method, there is no obvious spectral line of the center frequency 170 Hz in the spectrum, and many interference noises appear in the high-frequency part. Although the center frequencies of the simulated signal are obtained through the EEMD-WTD method, lots of noise still appeared in the spectrum and time domain waveform of the denoising signal.

To quantitatively evaluate the noise reduction performance of the denoising methods, the root mean squared error (RMSE) and the peak signal-to-noise ratio (PSNR) are used as the evaluation factors. The corresponding calculation formulae of PSNR and PMSE are described as follows:

(18) $$\left\{\begin{array}{l} RMSE=\sqrt{\frac{1}{N}\sum_{t=1}^{N}{\left[x(t)-x^{\prime }(t)\right]}^{2}}\\ PSNR=10\cdot \mathrm{lg}\left(\frac{255^{2}}{RMSE^{2}}\right) \end{array}\right.$$

where *x*(*t*) is the original signal, *x*′(*t*) is the denoised signal, and *N* is the length of the signal.

The calculation results of the evaluation factors of the denoising effect of different methods are shown in Table 5.

Compared with WTD, EMD-RD, and EEMD-WTD, the evaluation factors of the denoising signal by using VMD-MODWPT are better than other three methods. Combined with the analysis in Figure 12, Figure 13, Figure 14 and Figure 15, the effectiveness of the proposed method is proven.

The denoising evaluation factors of RMSE and PSNR from the above four methods for the simulated signal *z*(*t*) with different SNRs from −20 dB to 10 dB obtained by using the ‘awgn’ function in MATLAB software are shown in Table 6.

As shown in Table 6, the proposed VMD-MODWPT method can denoise the signals effectively. Compared with WTD, EMD-RD, and EEMD-WTD, VMD-MODWPT shows the best performance of RMSE and PSNR at different SNRs.

## 5. Experimental Case Study

Gears are installed in different kinds of machinery. Several problems of these machines may be caused due to defects within them. Generally, in the fault diagnosis of rotating machines, the signals to be analyzed often come with a lot of noise. To show the efficiency of the proposed method, in this section, the gear fault signal, which is mainly used to simulate the related faults of common mechanical equipment, is selected for analysis.

**Figure 16:**
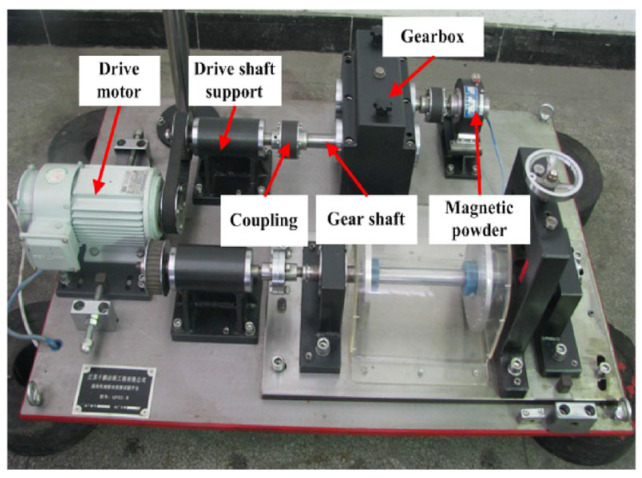
Gear experimental set-up.

The test apparatus used in this study is shown in Figure 16. The type of gear tester used is a QPZZ-II and the experiment set-up consists of a single-stage gearbox driven by a 0.75 kW AC governor motor. The driving gear consists of 55 teeth and the driven gear of 75 teeth, respectively. The signal monitoring and collection system consists of a KD1001L accelerometer, an amplifier, a data collector, and a computer. The sampling frequency is 5120 Hz. After sampling, the collected vibration signals are loaded into MATLAB from the data files.

During the test, the driving motor’s rotating frequency is 50 Hz. The acceleration sensor is installed on the bearing seat of the gearbox. The tape of the data acquisition card is ADA16–8/2 (LPCI). Each vibration signal consists of 5120 data points and the sampling precision is 16 bt. The parameters of the gearbox are shown in Table 7.

**Figure 17:**
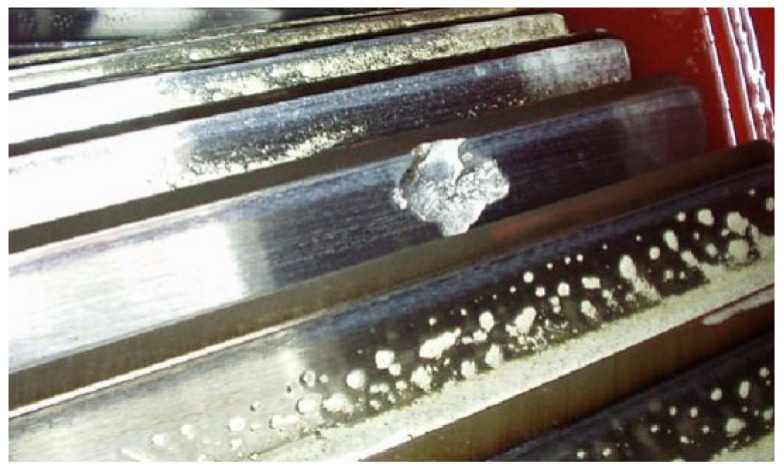
The driving gear with pitting fault.

The pitting fault is the cause of the fault of the driving gear. Figure 17 shows the driving gear; the main cause of this fault is the spot welding on the gear tooth surface. It makes the welding slag peel off, then a pit is formed on the tooth surface.

**Figure 18:**
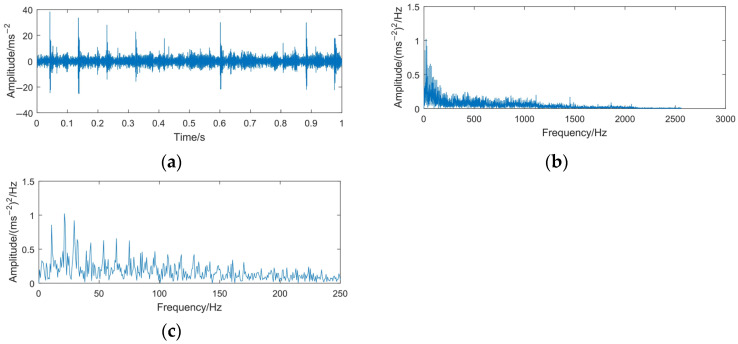
Time domain waveform and envelope spectrum of the gear pitting fault signal. (**a**) Waveform of the gear pitting fault signal; (**b**) envelope spectrum of the gear pitting fault signal; (**c**) envelope spectrum limited to 250 Hz of the gear pitting fault signal.

For gear fault diagnosis, envelope spectrum analysis is the most commonly used method. Therefore, it is necessary to resolve the envelope spectrum of the gear fault signal to diagnose the type of gear fault. The time domain waveform and envelope spectrum of the gear fault signal are shown in Figure 18.

It can be seen in Figure 18a,b that because no corresponding noise cancellation device is used in the signal acquisition system, the collected time domain signal contains more noise components. The maximum envelope spectrum is focused in 0–300 Hz of the frequency band. For ease of analysis, the envelope spectrum limited to 250 Hz is shown in Figure 16c. However, the fault characteristics and fault types of gears can be read and distinguished with difficulty; the main reason for the above problems is the influence of the noise interference components.

**Figure 19:**
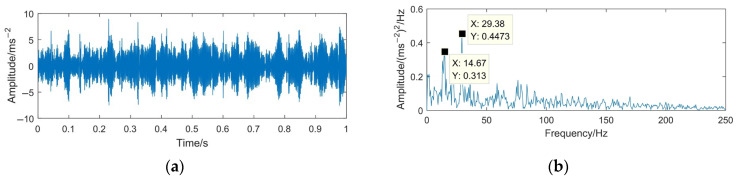
Time domain waveform and envelope spectrum of denoised signal based on VMD-MODWPT. (**a**) Waveform of the denoised signal; (**b**) envelope spectrum limited to 250 Hz of the denoised signal.

The time domain waveform and envelope spectrum of the denoised signal using the proposed VMD-MODWPT method are shown in Figure 19.

It can be seen from Figure 19 that most of the useless interference and noise components are filtered out effectively. In the envelope spectrum of the gear pitting fault denoising signal, 14.67 Hz and 29.38 Hz have obvious spectral lines, and 14.67 Hz and 29.38 Hz are about one and two times the rotating frequency of the driving gear. According to the principle of gear vibration [47], it is known that when a gear has a pitting fault, the rotating frequency and frequency doubling components of the shaft where the faulty gear is located can be obtained through envelope spectrum analysis, so it can be determined that the driving gear has a pitting fault.

**Figure 20:**
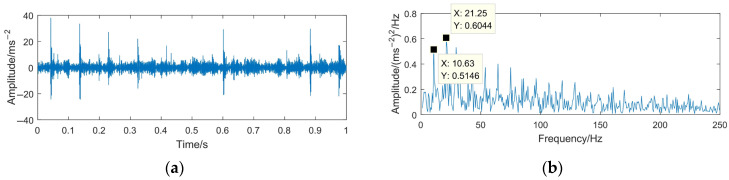
Time domain waveform and envelope spectrum of denoised signal based on WTD. (**a**) Waveform of the denoised signal; (**b**) envelope spectrum limited to 250 Hz of the denoised signal.

**Figure 21:**
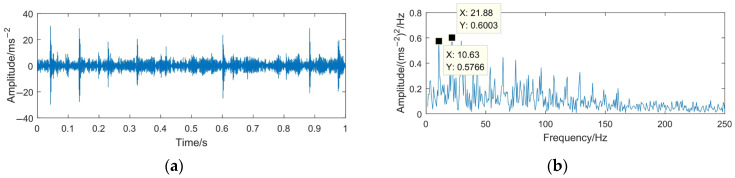
Time domain waveform and envelope spectrum of denoised signal based on EMD-RD. (**a**) Waveform of the denoised signal; (**b**) envelope spectrum limited to 250 Hz of the denoised signal.

**Figure 22:**
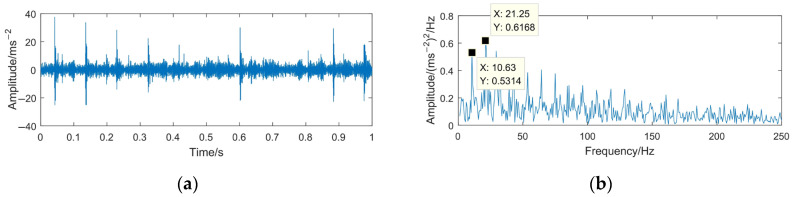
Time domain waveform and envelope spectrum of denoised signal based on EEMD-WTD. (**a**) Waveform of the denoised signal; (**b**) envelope spectrum limited to 250 Hz of the denoised signal.

To highlight the effectiveness and superiority of the proposed method, the WTD method, EMD-RD method, and EEMD-WTD method are used to deal with the gear pitting fault signal. The denoising results of the different methods are shown in Figure 20, Figure 21 and Figure 22. Based on [13,43,44,46], WTD adopts the ‘db3’ wavelet basis function for three-layer decomposition and the ‘Rigrsure’ rule for soft threshold denoising; for EEMD-WTD, an ensemble size of *I* = 100 and standard deviation *ε*_0_ = 0.2 are used in EEMD.

In Figure 20, Figure 21 and Figure 22, due to the influence of the noise and mode mixing problem, there are many interfering frequency components in the envelope spectra of the denoising signal obtained using WTD, EMD-RD, and EEMD-WTD. The spectral line features of 14.67 Hz and 29.38 Hz are not obvious, but 10.63 Hz and 21.25 Hz are clearly observed, which would reduce the accuracy of the fault feature extraction and the reliability of fault diagnosis, with 10.63 Hz being close to the rotating frequency of the driven gear (10.76 Hz). It also means that the completeness and denoising effect of the proposed method are much better than the other three decomposition methods.

**Figure 23:**
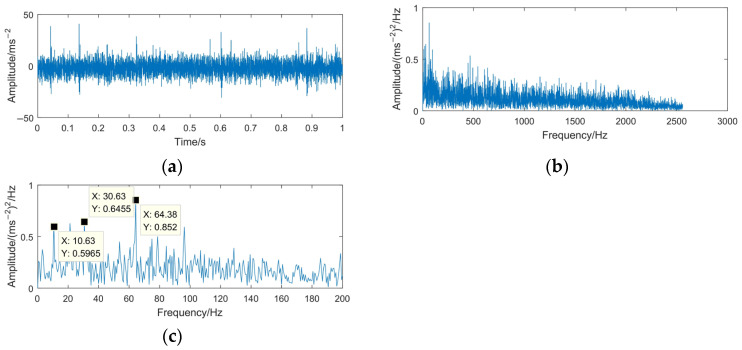
Time domain waveform and envelope spectrum of the gear pitting fault signal with strong noise. (**a**) Waveform of the gear pitting fault signal with strong noise; (**b**) envelope spectrum of the gear pitting fault signal with strong noise; (**c**) envelope spectrum limited to 200 Hz of the gear pitting fault signal with strong noise.

In order to verify the capability of the VMD-MODWPT method for dealing with the rotating machinery signals with strong noise, a Gaussian noise signal with −15 dB of SNR is added to the gear pitting fault signal, which is obtained by using the ‘awgn’ function in MATLAB software. Its time domain waveform and envelope spectrum are shown in Figure 23.

It can be seen from Figure 23 that the impulsive components in the time domain waveform are submerged by the noise. The envelope spectrum in the whole frequency band does not have obvious characteristics. The maximum envelope spectrum is focused in the 0–200 Hz frequency band. However, in this frequency band, it is also difficult to identify the fault characteristics.

The VMD-MODWPT method is used to denoise the gear pitting fault signal with strong noise. The time domain waveform and envelope spectrum of the denoised signal are shown in Figure 22.

**Figure 24:**
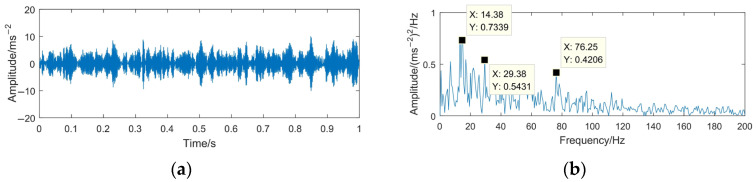
Time domain waveform and envelope spectrum of denoised signal based on VMD-MODWPT. (**a**) Waveform of the denoised signal; (**b**) envelope spectrum limited to 250 Hz of the denoised signal.

Comparing the time domain waveform and envelope spectrum of the denoised signal in Figure 24 with those of Figure 23, it can be seen that the impulsive components are obvious in the denoised signal, and 14.38 Hz, 29.38 Hz, an d76.25 Hz have obvious spectral lines in the envelope spectrum, which are the rotating frequency of the driving gear and multiples of its frequency. Therefore, under the condition of strong noise, it is able to effectively diagnose the pitting fault of gears.

**Figure 25:**
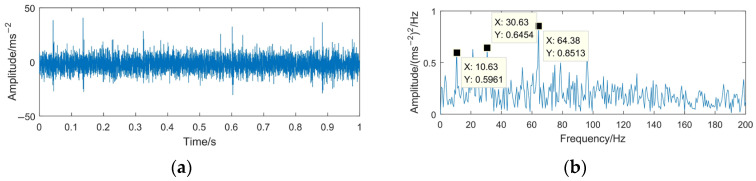
Time domain waveform and envelope spectrum of denoised signal based on WTD. (**a**) Waveform of the denoised signal; (**b**) envelope spectrum limited to 200 Hz of the denoised signal.

**Figure 26:**
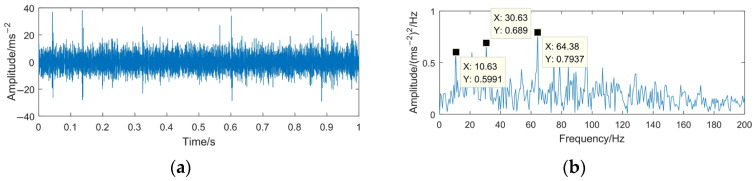
Time domain waveform and envelope spectrum of denoised signal based on EMD-RD. (**a**) Waveform of the denoised signal; (**b**) envelope spectrum limited to 200 Hz of the denoised signal.

**Figure 27:**
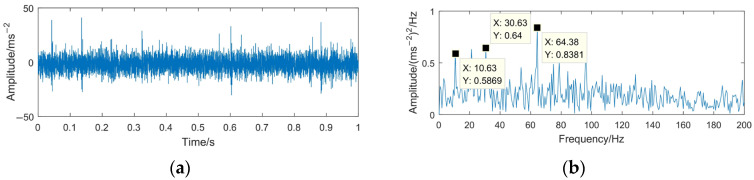
Time domain waveform and envelope spectrum of denoised signal based on EEMD-WTD. (**a**) Waveform of the denoised signal; (**b**) envelope spectrum limited to 200 Hz of the denoised signal.

The gear pitting fault signal with strong noise is also decomposed with the WTD method, the EMD-RD method, and the EEMD-WTD method. The denoised results are shown in Figure 25, Figure 26 and Figure 27. Based on [13,44,45,46], WTD adopts the ‘db3’ wavelet basis function for three-layer decomposition and the ‘Rigrsure’ rule for soft threshold denoising; for EEMD-WTD, an ensemble size of *I* = 100 and standard deviation *ε*_0_ = 0.2 are used in EEMD.

As can be seen from Figure 25, Figure 26 and Figure 27 the noise in the signal has not been effectively filtered, which means some impulsive components are hidden by the noise. Because of lots of interfering frequency components in the envelope spectrum of the denoised signal, the spectral line features of 14.67 Hz and 29.38 Hz are not obvious. The results proved that the completeness and denoising effect of the proposed method are much better than the other three decomposition methods, even for the signal with strong noise.

In order to quantify the evaluation of the denoising effect, the RMSE and PSNR are calculated for the four methods. The results are shown in Table 8 (for convenience of calculation, the collected gear pitting fault signal is treated as the clear signal). The results show that even for the signal with strong noise, the proposed VMD-MODWPT method can denoise with better performance.

## 6. Conclusions

This paper proposes a signal denoising method based on a fault feature of rotating machinery. The principle of minimum MDE and the number of effective centers *N* are proposed as parameters to be used in the selection of parameters for the signal. The CWE value is used to calculate the components that represent decomposition errors or background signals. The components with high frequencies are denoised and reconstructed. The denoised signal is reconstructed with sensitive and high-frequency components. The proposed method has advanced denoising performance when analyzing both simulation and real gear pitting fault signals, regardless of whether the signal has a high or low signal-to-noise ratio.

In this paper, the rotating machinery signal is investigated. It is thought that the method described in this paper performs better for other signals. In the future, we might examine the power system signal using this approach and evaluate it against other signal denoising methods. In addition, we will also try to study whether the proposed method is suitable only for steady states or whether it can also handle transitions or dynamic behaviour of rotary machinery.

## Figures and Tables

**Table 1:** The characteristics of common signal decomposition methods.

Decomposition Method	Characteristic	
	Advantages	Disadvantages
WT	Multi-resolution analysis.	Non-adaptive.
FastICA	(1) High computational efficiency; (2) Stable calculation process.	(1) Residual noise; (2) Difficulty in selecting denoising signals.
EMD	(1) Adaptive; (2) Completeness.	(1) End effect; (2) Mode mixing; (3) Illusive IMF.
EEMD	(1) Suppress modal mixing; (2) Adaptive.	(1) End effect; (2) Noise in IMF component; (3) Illusive IMF.
CEEMD	(1) Suppress modal mixing; (2) Adaptive; (3) High computational efficiency.	(1) End effect; (2) Noise in IMF component; (3) Illusive IMF.
VMD	(1) Suppress modal mixing; (2) Suppress end effect; (3) Better noise robustness.	(1) Key parameter selection; (2) Illusive IMF.
MODWPT	Translation invariance of wavelet coefficients and scale coefficients.	Unreasonable decomposition effect of fast-changing signals.

**Table 2:** The decision accuracy of each IMF component corresponding to different *K* values.

*K* Value	The Decision Accuracy of Each IMF Component *ε_K_*_,*i*_					
	*ε_K_*_,1_	*ε_K_*_,2_	*ε_K_*_,3_	*ε_K_*_,4_	*ε_K_*_,5_	*ε_K_*_,6_
*K* = 2	0.0240	0.4009	−	−	−	−
*K* = 3	0.0123	0.0219	0.2795	−	−	−
*K* = 4	0	0.0056	0.0183	0.0060	−	−
*K* = 5	0.0560	0.2650	0.3732	0.3858	0.2380	−
*K* = 6	0.0066	0.1535	0.0035	0.0087	0.0293	0.1233

**Table 3:** The decision accuracy of each IMF component of the bearing inner ring fault signal.

*K* Value	The Decision Accuracy of Each IMF Component *ε_K_*_,*i*_				
	*ε_K_*_,1_	*ε_K_*_,2_	*ε_K_*_,3_	*ε_K_*_,4_	*ε_K_*_,5_
*K* = 2	0	0.2706	−	−	−
*K* = 3	0.5336	0.4937	0.2706	−	−
*K* = 4	0	0	0	0.0452	−
*K* = 5	0	0	0	0.1591	0.0452

**Table 4:** The DID value of IMF3 components decomposed through MODWPT.

Component	DID
C1	8.2391
C2	0.1823
C3	1.5912
C4	8.3630

**Table 5:** Comparison of denoising effect evaluation factors of different methods.

Denoising Method	Evaluation Factor	
	RMSE	PSNR
WTD	0.1668	55.9080
EMD-RD	0.8637	48.7671
EEMD-WTD	0.9741	48.2449
VMD-MODWPT	0.1650	55.9564

**Table 6:** Denoising evaluation factor with different SNRs (dB).

SNR/dB	Denoising Method	Evaluation Factor	
		RMSE	PSNR
−20	WTD	93.3649	28.4290
	EMD-RD	75.3798	29.3583
	EEMD-WTD	94.3911	28.3815
	VMD-MODWPT	18.4344	35.4745
−15	WTD	30.4595	33.2936
	EMD-RD	24.8190	34.1830
	EEMD-WTD	31.6566	33.1262
	VMD-MODWPT	5.6198	40.6336
−5	WTD	2.0354	45.0443
	EMD-RD	2.5859	44.0047
	EEMD-WTD	3.1486	43.1486
	VMD-MODWPT	0.6412	50.0609
5	WTD	0.0833	58.9259
	EMD-RD	0.3269	52.9861
	EEMD-WTD	0.3076	53.2509
	VMD-MODWPT	0.0731	59.4889
10	WTD	0.0566	60.6056
	EMD-RD	0.1597	56.0988
	EEMD-WTD	0.1078	57.8038
	VMD-MODWPT	0.0337	62.8599

**Table 7:** The gearbox parameters.

Parameters	Numerical Values
Gear material	S45C
Number of teeth of driving gear	55
Number of teeth of driven gear	75
Module of gear/mm	2
Average speed of input shaft/rpm	880
Rotating frequency of driving gear/Hz	14.67
Average speed of output shaft/rpm	629
Rotating frequency of driven gear/Hz	10.76
Meshing frequency/Hz	806.67

**Table 8:** Comparison of denoising effect evaluation factors of different methods.

Denoising Method	Evaluation Factor	
	RMSE	PSNR
WTD	31.9753	33.0827
EMD-RD	34.0391	32.8110
EEMD-WTD	32.9687	32.9498
VMD-MODWPT	12.8307	37.0483

## Data Availability

Not applicable.
